# Promising dawn in the management of pulmonary hypertension: The mystery veil of gut microbiota

**DOI:** 10.1002/imt2.159

**Published:** 2024-01-01

**Authors:** Yicheng Yang, Hanwen Zhang, Yaoyao Wang, Jing Xu, Songren Shu, Peizhi Wang, Shusi Ding, Yuan Huang, Lemin Zheng, Yuejin Yang, Changming Xiong

**Affiliations:** ^1^ State Key Laboratory of Cardiovascular Disease, Department of Cardiology Fuwai Hospital, National Center for Cardiovascular Diseases, Chinese Academy of Medical Sciences and Peking Union Medical College Beijing China; ^2^ State Key Laboratory of Cardiovascular Disease, Department of Nephrology Fuwai Hospital, National Center for Cardiovascular Diseases, Chinese Academy of Medical Sciences and Peking Union Medical College Beijing China; ^3^ Department of Genetics University Medical Center Groningen, University of Groningen Groningen The Netherlands; ^4^ State Key Laboratory of Cardiovascular Disease, Department of Cardiac Surgery Fuwai Hospital, National Center for Cardiovascular Diseases, Chinese Academy of Medical Sciences and Peking Union Medical College Beijing China; ^5^ Center for Molecular Cardiology University of Zurich Zurich Switzerland; ^6^ China National Clinical Research Center for Neurological Diseases, Tiantan Hospital, Advanced Innovation Center for Human Brain Protection The Capital Medical University Beijing China; ^7^ Key Laboratory of Molecular Cardiovascular Sciences of Ministry of Education, NHC Key Laboratory of Cardiovascular Molecular Biology and Regulatory Peptides, School of Basic Medical Sciences, Health Science Center The Institute of Cardiovascular Sciences and Institute of Systems Biomedicine, Peking University Beijing China

**Keywords:** disease management, gut–lung axis, gut microbiota, metabolites, pulmonary hypertension

## Abstract

The gut microbiota is a complex community of microorganisms inhabiting the intestinal tract, which plays a vital role in human health. It is intricately involved in the metabolism, and it also affects diverse physiological processes. The gut–lung axis is a bidirectional pathway between the gastrointestinal tract and the lungs. Recent research has shown that the gut microbiome plays a crucial role in immune response regulation in the lungs and the development of lung diseases. In this review, we present the interrelated factors concerning gut microbiota and the associated metabolites in pulmonary hypertension (PH), a lethal disease characterized by elevated pulmonary vascular pressure and resistance. Our research team explored the role of gut‐microbiota‐derived metabolites in cardiovascular diseases and established the correlation between metabolites such as putrescine, succinate, trimethylamine N‐oxide (TMAO), and N, N, N‐trimethyl‐5‐aminovaleric acid with the diseases. Furthermore, we found that specific metabolites, such as TMAO and betaine, have significant clinical value in PH, suggesting their potential as biomarkers in disease management. In detailing the interplay between the gut microbiota, their metabolites, and PH, we underscored the potential therapeutic approaches modulating this microbiota. Ultimately, we endeavor to alleviate the substantial socioeconomic burden associated with this disease. This review presents a unique exploratory analysis of the link between gut microbiota and PH, intending to propel further investigations in the gut–lung axis.

## Highlights

- The significant development in gut microbiota has brought an unprecedented paradigm for pulmonary hypertension (PH) management in recent years, which has delighted researchers and clinicians in precise disease control.
- The gut–lung axis is the bidirectional interaction between the gut and lung, which occurs through circulation, the nervous system, and other physiological processes, ultimately affecting the health of both the gut and the lung.
- We provided a comprehensive landscape of gut microbiota and its associated metabolites in PH, emphasizing the interplay between microorganisms and hosts in modulating PH.
- With the rapid development of technologies, including metagenomic and metabolomics on gut microbiota, the association of microorganisms and the human body will be untangled eventually, which will bring the dawn for disease cure followed by prognosis improvement among patients with PH.

## Graphical abstract

With the rapid development of technologies, including metagenomic and nontargeted or targeted metabolomics, the knowledge of gut microbiota and their related metabolites is constantly growing. Dysfunctions of microorganisms are associated with cardiovascular diseases, and gut microbiota and its associated metabolites are also involved in the pathogenesis of pulmonary hypertension. In addition, pulmonary hypertension might result in the alteration of bacterial flora. Here, we provide a comprehensive landscape of gut microbiota and metabolites in pulmonary hypertension, emphasizing the interplay between microorganisms and hosts in modulating pulmonary hypertension.

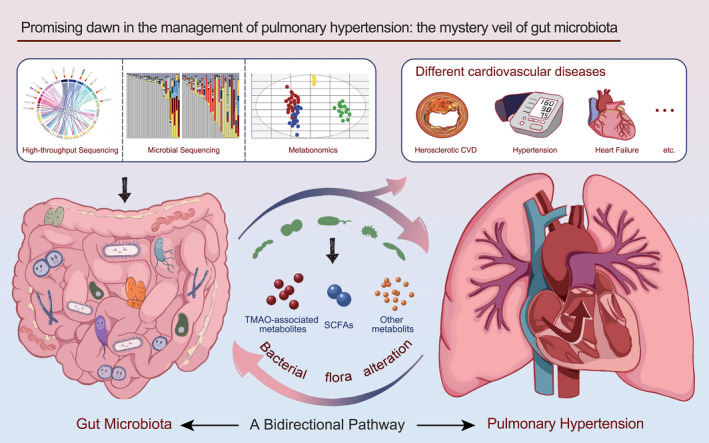

## INTRODUCTION

Pulmonary hypertension (PH) is a pathophysiological state resulting from various pathogenetic factors. As per the 2022 European Society of Cardiology/European Respiratory Society guideline, PH is defined as an average pressure of greater than 20 mmHg in mean pulmonary artery pressure on supine right heart catheterization at rest [1]. PH is a progressive disease that can lead to heart failure, ultimately leading to fatality. With the advancement in disease and medical examination technology, PH has gained increased attention as a cardiovascular disease (CVD) in the past decade. PH patients present with unspecific signs and symptoms, including fatigue, exertional dyspnea, chest pain, edema of the legs or abdomen, and dizziness. Hence, when diagnosed, most PH has deteriorated to the advanced stage and missed the optimal treatment timing, leading to short survival time and poor prognosis. Based on pathogenetic causes and relevant treatments, PH has been categorized into Groups 1−5, including pulmonary arterial hypertension (PAH), PH associated with left heart disease, PH associated with lung diseases and/or hypoxia, PH associated with pulmonary artery obstructions, and PH with unclear and/or multifactorial mechanisms [1, 2, 3, 4].

PAH is a condition distinguished by elevated pulmonary vascular pressure and resistance, which is caused by the proliferative, exogenic, and fibrotic remodeling of pulmonary arterioles [2, 5, 6]. While the previous perspectives attributed the abnormality of the pulmonary vessels to the overproliferated smooth muscle cells and dysfunctional endothelial cells, recent research has shifted the focus toward the modulation of interstitial cells and immuno‐inflammatory cells [7, 8, 9], as well as metabolic dysfunction (Warburg effect) in PAH [10, 11, 12]. Despite our current in‐depth understanding of PAH, clinical treatment still relies merely on targeted drugs, including phosphodiesterase‐5 inhibitors, endothelin receptor antagonists, soluble guanylate cyclase stimulators, and prostaglandins. Disappointingly, these drugs can not reverse the pathogenesis of pulmonary vascular remodeling, leading to the failure to prevent deterioration and the need for a lung transplant in severe cases [2]. For another subtype of Group 4 PH named chronic thromboembolic pulmonary hypertension (CTEPH), therapeutic options such as balloon pulmonary angioplasty and pulmonary endarterectomy are regarded as clinically beneficial strategies [13, 14, 15, 16]. However, the invasive injury, complexity of operations, and time‐consuming nature hinder prompt interventions on the disease. Despite our current understanding of PH, it is still a great challenge to find effective therapies for this “cancer‐like” disease. Further exploration of treatment paradigms is essential to achieve the real “cure” of PH.

The significant development in gut microbiota has brought an unprecedented paradigm for disease management in recent years, which has delighted researchers and clinicians in precise disease control [17, 18]. The intestinal system is the largest organ in the human body. It contains a vast community of microorganisms, with bacteria, archaea, fungi, and viruses being the predominant members constituting the unique gene pool in each human body. Animal species, including humans, co‐evolve with microbial communities [18, 19, 20]. Microorganisms constantly adapt to their hosts' environments and ecologies in the process of nutrient absorption [19], colonization resistance [21], and immunity [22]. Gut microbiota composition and its related nucleic acid and metabolites interact with human functions in a fine‐tuned bidirectional relationship to influence individual health [23, 24] With the development in high‐throughput sequencing and metagenomics, we can obtain information on dynamic changes in human gut microbiota, thus helping to unveil the mystery of gut microorganisms. In the past decade, gut microbiota in humans has captured the sustained interest of researchers, and its involvement in the pathogenesis of CVDs has been elucidated. For example, increased abundance of *Enterobacteriaceae* and *Streptococcus* spp. resulted in the phenotype of atherosclerotic CVD [25]. Compared with the healthy populations, decreased microbial richness and diversity accompanied by the excessive growth of bacteria such as *Prevotella* and *Klebsiella* were discovered in hypertension patients [26]. In addition, patients with heart failure exhibited decreased microbial richness, including depletion of taxa in the Lachnospiraceae family [27]. Although not as thoroughly studied as gut microbiota in other CVDs, quite a little progress has been made in microorganisms in PH. In particular, the landscape of altered gut microbiome in PAH patients has been portrayed as characterizing increased trimethylamine (TMA)/trimethylamine N‐oxide (TMAO)‐producing bacteria and decreased butyrate‐ and propionate‐producing bacteria, including *Coprococcus*, *Butyrivibrio*, *Eubacterium*, *Akkermansia*, *Bacteroides*, and *Lachnospiraceae* [28].

However, limited by the significant heterogeneity among individuals, uncovering the actual variation of gut microbiota and their accurate role in disease pathogenesis is a considerable challenge. The products derived from gut microbiota, particularly its related metabolites, seem to be another window for catching sight of the accurate interaction between microorganisms and diseases. Mounting evidence has clarified the association between CVDs and metabolites, including TMA‐related products, short‐chain fatty acids (SCFAs), bile acids, and amino acids [29, 30, 31, 32]. Our research team has investigated the involvement of gut‐microbiota‐derived metabolites in CVDs and has demonstrated the association between putrescine [33], succinate [34], TMAO [35, 36, 37], and N, N, N‐trimethyl‐5‐aminovaleric acid (TMAVA) [38, 39] with CVDs. Moreover, we found that metabolites, such as TMAO and betaine, were potential biomarkers in the area of PH, showing significant clinical value [40, 41].

**Figure 1:**
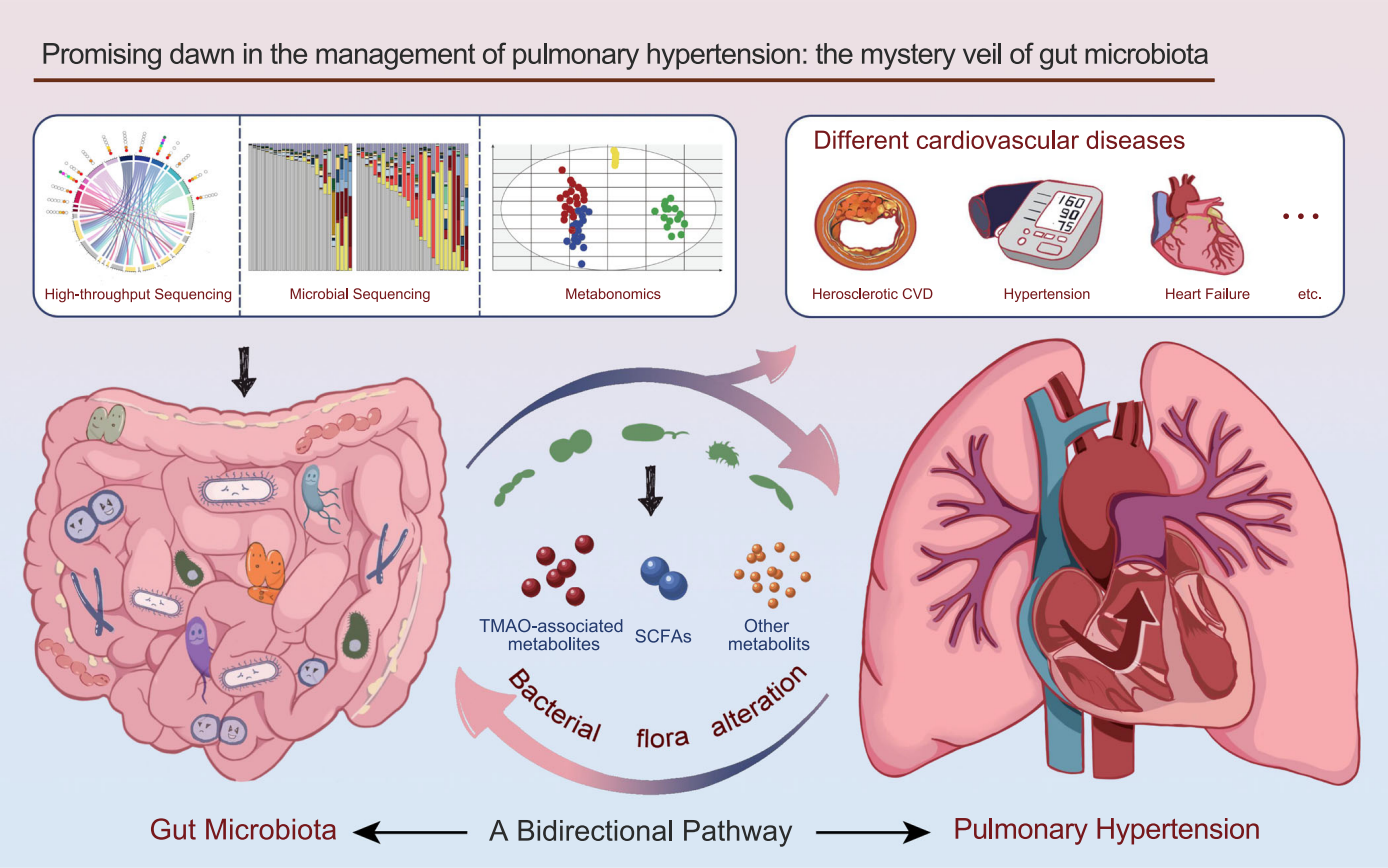
Presentations of the association between gut microbiota and pulmonary hypertension. With the rapid development of technologies, including metagenomic and nontargeted or targeted metabolomics, the knowledge of gut microbiota and their related metabolites is constantly growing. Dysfunctions of microorganisms are associated with cardiovascular diseases, including heart failure, hypertension, and atherosclerotic heart disease. Recently, it has been demonstrated that gut microbiota and its associated metabolites are also involved in the pathogenesis of pulmonary hypertension. In addition, pulmonary hypertension might result in the alteration of bacterial flora. Here, we provide a comprehensive landscape of gut microbiota and metabolites in pulmonary hypertension, emphasizing the interplay between microorganisms and hosts in modulating pulmonary hypertension. CVD, cardiovascular disease; SCFA, short‐chain fatty acid; TMAO, trimethylamine N‐oxide.

With the rapid development of technologies, including metagenomic and nontargeted or targeted metabolomics on gut microbiota, the intricate relationship between microorganisms and the human body will be deciphered over time, which will bring the dawn for disease cure followed by prognosis improvement among patients with PH (Figure 1). To date, more studies have focused on the microbiota and its related products in PH, devoted to changing the present defective management paradigms. This review comprehensively presents the landscape of gut microbiota and its associated metabolites in PH, emphasizing the interplay between microorganisms and hosts in modulating PH. We believe our work can promote the development of PH, ultimately contributing to reducing the disease burden.

## GUT–LUNG AXIS

The gut–lung axis delineates the bidirectional communication between the gut and lung, shaped by environmental factors, gut microbiota, and metabolites. This interaction occurs through circulation, the nervous system, and other physiological processes, ultimately affecting the health of both the gut and the lungs [42]. The gut–lung axis concept was initially introduced by Turner–Warwick in 1968 [43], which led to subsequent investigations into the correlation between lung and gut diseases. In 1976, Kraft et al. observed a higher incidence of chronic bronchopulmonary disease among individuals with severe inflammatory bowel disease [44]. CT scans unveiled an association between the severity of gut and lung symptoms [44], suggesting that gut damage and translocation of lipopolysaccharide (LPS) into the circulation could lead to lung injury. This link between the gut–lung axis and spontaneous bone marrow transplant complications has also been established [45]. Additionally, lung diseases have demonstrated an impact on the gut microbiota and increase the risk of intestine dysfunction, as asthma patients had twice the incidence of irritable bowel syndrome as healthy individuals [46]. While the gut–lung axis has been confirmed in human and mouse models, the underlying mechanisms remain unclear. Continued discourse on this subject will enhance our comprehension of the interplay between gut microbiota and PH.

### Homology between lungs and intestines

The homology between lungs and intestines is the structural basis for the “gut–lung axis.” The lung, trachea, respiratory epithelium, and intestines have homology in embryonic development, all originating from the endoderm [47]. A recent study showed that changing the activity of transcription factors induced intestinal progenitor cells from embryonic lung progenitor cells, which can further produce various types of intestinal cells [48]. The respiratory and digestive mucosa belong to the mucosal immune system, both having a typical carrier system and can produce large amounts of secretory immunoglobulin A (sIgA). sIgA is a common molecular basis for mucosal immunity in various sites and a vital substance that reflects the “lung–gut axis” [49]. Many laboratories have already proven that sIgA can prevent pathogenic bacteria from spreading within the host tissue and can bind to many nonpathogenic or beneficial bacteria [50]. These discoveries imply a noteworthy involvement of this particular antibody in regulating the composition of microbial communities. In addition, researchers found that the lung functional protein pulmonary surfactant protein A can be detected in the intestinal tissue of patients with intestinal inflammation [51]. The chemokine eotaxin, which initially recruits eosinophils to the site of the lesion, and its mRNA can be expressed in both the lungs and intestines [52, 53]. The many similarities in structure and immune regulatory mechanisms between the intestinal and lung mucosa form the biological basis for bidirectional communication within the gut–lung axis.

### The immune response

The mucosal immune system involves the lungs and the intestine, and inflammation in one organ may reflect in the other. Micro‐fold cells in mucosa‐associated lymphoid tissue recognize antigens and present them to dendritic cells. These dendritic cells then migrate to lymph nodes and stimulate immune responses from T and B lymphocytes when pathogens are present [54]. The lungs and intestines share structural and functional similarities, allowing for the presence of common homing receptors, such as CC‐chemokine receptor (CCR) 6, α4β7, and CCR9. These receptors recruit T cells to the intestine, while CCR4 directs T cells to the lungs [55]. The interaction of adhesion molecules, specifically VCAM‐1 and α4β1 integrin, along with L‐selectin and PNAd, is essential for facilitating immune cell homing to the lungs. During intestinal inflammation, T effector memory cells are generated and express L‐selectin/CD62L. When these cells enter the circulation, they bind to the PNAd ligand on the lung endothelium [56]. Lymphocytes rely on these receptors to move between organs in the periphery. An in vitro study demonstrated the ability of lung dendritic cells to induce the expression of CCR9 and α4β7 on T cells, providing evidence for an entero‐pulmonary axis. When a mucosal lesion occurs, it generates an immune response that may affect other mucosal immune responses through the mucosal immune pathway. This can lead to varying degrees of immune response in local stimulation at different mucosal sites. For instance, intestinal microorganisms have the capacity to stimulate the generation of type 2 and type 3 intrinsic lymphocytes, which migrate through lymph and blood circulation to the respiratory tract, potentially causing damage [55].

### Metabolites of gut microbiota

The gut–lung axis involves various factors that contribute to its functional roles, and one of the indispensable mediators is the gut metabolites. Metabolites generated by gut microbiota circulate in the bloodstream, facilitating bidirectional communication between the lungs and the gut and exerting distinct effects on each organ.

One well‐known illustration of these metabolites is SCFAs, such as butyrate, propionate, and acetate. A decrease in SCFA‐producing bacteria levels in the gut leads to reduced circulating levels of SCFAs [57]. Probiotic dietary fiber, which regulates gut microbiota, is not degraded by the digestive enzymes but is converted to SCFAs by gut microbiota [58]. Butyrate, as a representative SCFA, plays an essential role in gut epithelial cell function and promotes differentiation and maturation of immune cells, thereby regulating the gut immune system [59]. SCFAs also play a vital role in mucosal immunity, enhancing the metabolism of plasma B‐cells and increasing the permeability of tight junctions to promote differentiation of goblet cells and mucin production. This leads to enhanced production of intestinal IgA to strengthen the intestinal epithelial barrier function [60]. Furthermore, these SCFAs have anti‐inflammatory properties. They induce Treg activation via G protein‐coupled receptors or through epigenetic modifications by inhibiting histone deacetylases (HDACs) [61, 62]. The microbial synthesis of SCFAs facilitates the generation of T‐regulatory cells in the colon [63]. Interestingly, mice fed a high‐fiber‐fed exhibit higher circulating levels of SCFAs and show a preventive effect against allergic inflammation in the lungs. In contrast, mice fed a low‐fiber‐fed exhibit lower circulating levels of SCFAs and heightened susceptibility to allergic lung disease [64]. Notably, SCFAs not only regulate the gastrointestinal immune system but also influence the bacterial profile of the gastrointestinal tract. Butyrate induces peroxisome proliferator‐activated receptor‐γ, promoting a shift in colonic epithelium toward β‐oxidation and the preservation of an anaerobic environment favored by the obligate anaerobes in the colon [65]. Conversely, the absence of butyrate leads to elevated inducible nitric oxide synthase expression and Proteobacteria expansion. Proteobacteria, facultative anaerobes proficient in nitrate respiration, are typically more abundant in dysbiotic states [65]. Butyrate regulates lung disease pathogenesis by influencing the bacterial profile in the gastrointestinal tract. In summary, some SCFAs are utilized as an energy source by gut cells, while other SCFAs, such as butyrate, can cross the membrane, entering the peripheral blood system and influencing the differentiation and maturation of immune cells in the bone marrow cavity. Immune cells regulated by butyrate enter the lungs through blood circulation, regulating immune responses in the lungs [66].

TMAO is a byproduct of intestinal flora metabolism recognized as a potential risk factor for CVDs and other chronic illnesses, including lung diseases. Elevated concentrations of TMAO were demonstrated to be predictive of CVDs risk nearly a decade ago [67]. Recently, its association with pulmonary diseases has also been gradually revealed. TMAO mainly stems from choline, abundant in red meat and fish. Intestinal flora choline‐TMA lyase can break it down to produce TMA, which enters the liver via the portal vein and undergoes oxidation by flavin‐containing monooxygenases to generate TMAO ultimately [67, 68]. After synthesis in the liver, TMAO is transported through the bloodstream to various systems in the body, including the lungs and heart [69]. TMAO stimulates inflammation and causes structural damage to the pulmonary blood vessels. Studies have demonstrated that heightened blood concentrations of TMAO can impair endothelial function and trigger vascular inflammation due to increased oxidative stress [70, 71]. TMAO activates the TXNIP‐NLRP3 inflammasome, leading to endothelial nitric oxide synthase activity inhibition and reduced nitric oxide production. This process induces oxidative stress and elevates the levels of interleukin‐1β and interleukin‐18 [72]. TMAO enhances the transforming growth factor‐beta (TGF‐β)/Smad content in right ventricular fibroblasts when exposed to hypoxia. This growth factor engages its receptor and activates fibroblasts through the second messenger Smad, which stimulates the release of type I collagen and contributes to cardiac and pulmonary fibrosis. The increased expression of molecules related to the TGF‐β/p38 mitogen‐activated protein kinase (MAPK)/Smad pathway suggests their involvement in the onset of pulmonary and right ventricular fibrosis [73, 74].

In addition, the regulation roles of other gut microbiota‐related metabolites in the lung, including deaminated tryptophan [75]. are being discovered, which contributes to further knowledge of the gut–lung axis.

### Microbial components in the gut

Microbial components are another soluble substance that facilitates cross‐talk between the lungs and the gut via the circulatory system. Microbial components include microbial‐associated molecular patterns, which interact with host pattern recognition receptors, like toll‐like receptors (TLRs) or nod‐like receptors [76], to generate immune inflammatory effects in the human body. Microbial‐associated molecular patterns include peptidoglycans and LPSs, with LPS being a crucial constituent of the outer membrane of most gram‐negative bacteria, playing a critical role in protecting bacteria from environmental stress, antibiotic resistance, and symbiosis [77]. LPS is essential for human health and triggers a different mechanism of activating immunity compared to other immune agents, the only substance that activates pulmonary phagocytes through TLR4 [78]. After TLR4 activation, there are conformational changes within the cells, signal transmission within the cells, and activation of nuclear factor kappa‐B (NF‐κB) and MAPK pathways [79, 80]. These two pathways eventually release inflammatory responses, such as interleukin‐1, interleukin‐6, TNF‐α, and nitric oxide [81]. ultimately resulting in acute lung injury [82]. Research has shown that mice with cleared gut microbiota by antibiotics are more vulnerable to the influenza virus. The TLR7 signaling pathway is hindered, leading to a downregulated immune response and an impaired immune activity of certain T lymphocyte subpopulations, making it difficult for the influenza virus to be cleared [83, 84]. Excessive reactive oxygen species production is a significant factor in LPS‐induced acute lung injury [85]. The attack of reactive oxygen species on mitochondrial DNA in lung cells leads to cell death and tissue damage. Injury to alveolar epithelial cells and endothelial cells in the capillaries results in diffuse interstitial lung edema and acute hypoxemic respiratory failure [86].

### Dysbiosis of gut microbiota

Dysbiosis of gut microbiota is a visible manifestation of an imbalanced lung–gut axis, closely linked with developing respiratory diseases. The human intestine serves as a vast microbial habitat, with thousands of bacteria residing in the adult intestine, totaling up to 10^14^, nearly 10 times the count of adult cells and 100 times the count of genes encoded within the human body [87, 88]. Four bacterial phyla (Bacteroidetes, Firmicutes, Proteobacteria, and Actinobacteria) predominate in the human colon [89]. Alterations in the gut microbiota's composition and functions can influence the respiratory system via a shared mucosal immune system. Mucin secretion from the gut epithelium cells limits bacterial migration into epithelial tissue. Once the mucosal epithelium is damaged, microbial translocation occurs, leading to systemic inflammation followed by severe diseases such as sepsis [90]. Moreover, mucin and its components can shape microbiota residing in composition in the lumen of the mucosal tissue. This is achieved by some bacteria adhering, surviving, feeding on mucin, or altering the mucin layer [89].

It is crucial to emphasize the bidirectional nature of the gut–lung axis, as respiratory diseases frequently exhibit an imbalance in an imbalanced equilibrium between microbial immigration and elimination in the gut. For instance, patients with COVID‐19 exhibit dynamic changes in their gut microbiota throughout hospitalization, marked by a decline in SCFA‐producing bacteria (such as *Faecalibacterium*) and an elevation in facultatively anaerobic bacteria (such as *Escherichia–Shigella*) [91]. A consistent rise in *Eggerthella* belonging to the class Coriobacteriia was also noted, potentially contributing to increased intestinal permeability and subsequent gastrointestinal symptoms [92]. Given that a leaky gut induces translocation of intestinal bacteria, this could initiate systemic inflammation, culminating in the progression of severe disease. Aside from COVID‐19, specific bacteria are often detected in pathological fecal matter, such as *Bacteroides*, *Faecalibacterium*, *Agathobacter*, *Blautia A*, and *Roseburia* (in the case of asthma) or *Faecalicatena*, *Oscillibacter*, *Lawsonibacter*, *Flavonifractor*, and *Streptomyces* (in the case of chronic obstructive pulmonary disease [COPD]) [93]. A reduced α‐diversity of intestine flora could be detected in mycobacterium tuberculosis patients compared to controls, with a relatively increased abundance of Proteobacteria and a decreased abundance of Bacteroides [94, 95, 96]. Chronic inflammation‐induced changes in physicochemical properties can promote the establishment of specific species within the microbial community, transitioning them from transient to resident members. Many respiratory disorders have manifestations in the gastrointestinal tract and impact the composition of intestine flora. Influenza virus pneumonia induces diarrhea via an increase in the expression of lipid carrier protein‐2 and colonic mucin Muc5ac, indicating the occurrence of mild intestinal inflammation [97, 98]. Bacterial pneumonia caused by multidrug‐resistant *Pseudomonas aeruginosa* can induce gastrointestinal disorders, possibly by inhibiting the proliferation of gastrointestinal epithelial cells by restricting the cell cycle of the M phase [99]. CD4^+^T cells have a positive correlation with intestine flora to maintain the ecological balance [94]. Although some researchers have proposed that lung disease may have an impact on intestinal microbiota, the precise molecular mechanisms underlying the influence of the lungs on the intestinal flora remain poorly understood. Oxygen homeostasis is essential for the maintenance of gut flora function. The fetal lungs develop under low oxygen conditions, and premature birth can expose the developing lungs to a hyperoxia environment. A hyperoxia environment can directly inhibit the expression of antimicrobial peptides in intestinal epithelial cells and can also affect the composition of the ileal microbiota, particularly leading to an increase in *Staphylococcus* abundance. Antimicrobial peptides play a critical role in regulating the stability of the host gut microbiota. The damage to ileal epithelial cells resulting from hyperoxia exposure is associated with the downregulation of antimicrobial peptide expression [100]. On the contrary, hypoxia caused by COPD results in gastrointestinal integrity damage, and low active glucose transport and reduced protein digestion and absorption cause the disturbance in the intestinal flora along with the decrease in the synthesis of SCFAs and the concentration of acetic acid in serum, which reversely aggravates the severity of the disease [101]. Immunity is another one of the potential factors. Type 2 ILC (ILC2) in mice promotes intestinal mucosa antiparasite defense and tissue repair. These cells can be transferred from the lungs to the intestine and mature via the pulmonary‐intestinal axis during IL‐33 stimulation and postnatal developmental stages. Hyperactivation of allergen‐induced lung ILC2 can cause inflammation of gut ILC2, while the developmental defect of lung ILC2 can significantly affect the number and function of intestine ILC2 [102, 103]. Another potential pathogenesis involving the gut–lung axis is long noncoding RNA. Research demonstrates that miR‐155 and miR‐21 are significantly upregulated both in pulmonary and intestinal mucosal injury tissue. MiR‐155 targets SOCS1 in the lung and FOXO3a in the gut to play a role in proinflammation [104, 105], while miR‐21 promotes fibrosis via 5MAD7 in the lung and reduces intestinal barrier function via RhoB [106, 107, 108]. Although epigenetics does not reveal which is the cause and which is the effect of the gut and the lung, it suggests a new direction to explore the relationship between them. Recently, changes in respiratory microbial composition have been linked to the development of PH. Exposure to *Streptococcus salivarius* resulted in increased right ventricular systolic pressure, right ventricular hypertrophy, and pulmonary vascular remodeling, all typical characteristics of PH. *S. salivarius*‐induced PH was characterized by increased inflammation in the lung and was also associated with a shift in the gut microbiome composition, indicating potential communication between the lung and gut axis [109]. Overall, the specific impact of changes in lung microbiota on gut function is still poorly characterized and needs further research efforts.

**Figure 2:**
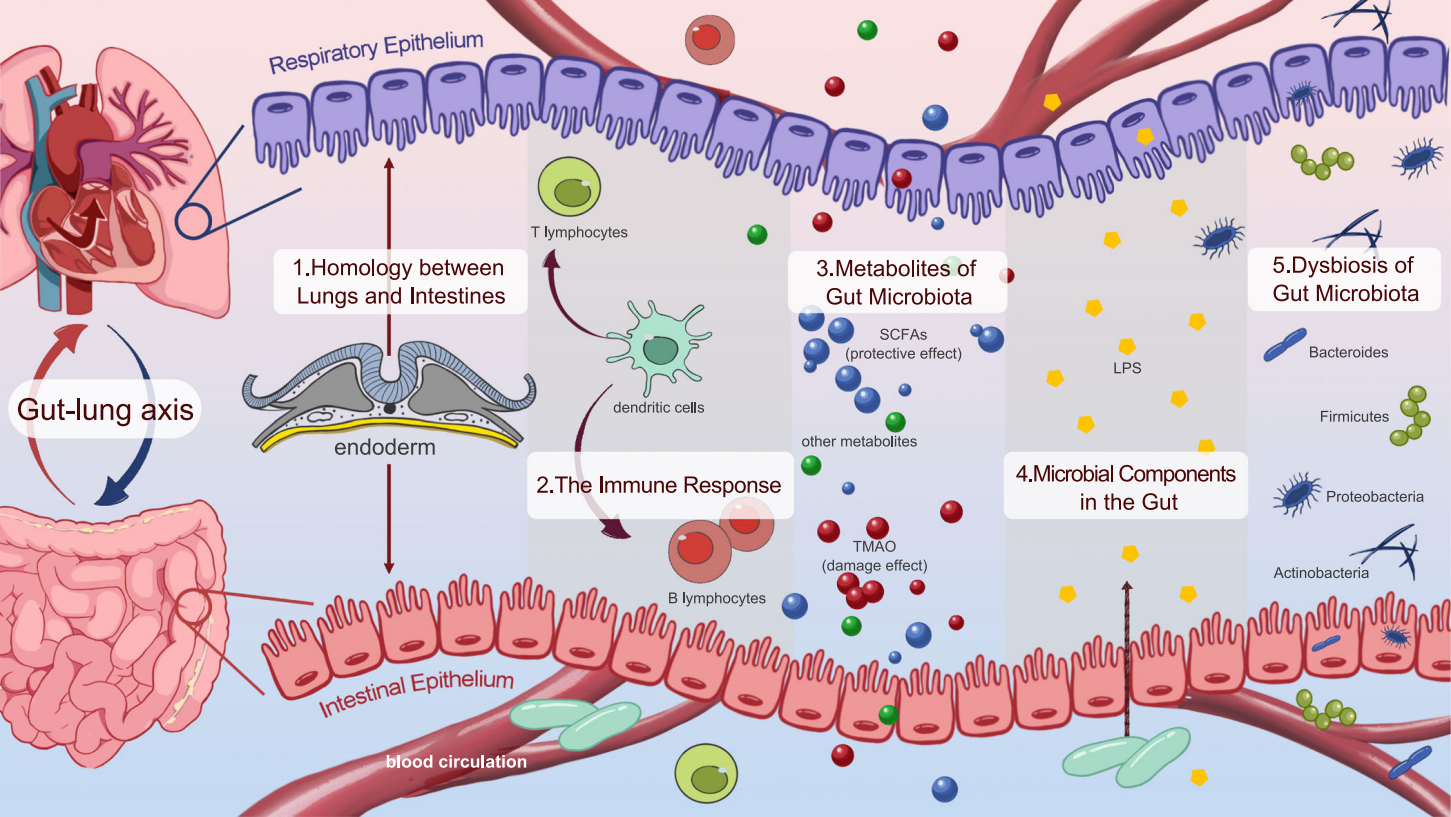
Bidirectional gut–lung axis. The gut–lung axis is the interaction between the gut and lung, which is influenced by environmental factors, gut microbiota, and metabolites. This interaction occurs through circulation, the nervous system, and other physiological processes, ultimately affecting the health of both the gut and the lungs. (1) The homology between the lungs and intestines is the structural basis for the gut‐lung axis. (2) Micro‐fold cells in muco‐associated lymphoid tissue recognize antigens and present them to dendritic cells, which migrate to lymph nodes and stimulate immune responses from T and B lymphocytes when pathogens are present. (3) Metabolites produced by gut microbiota, including TMAO and SCFAs, circulate through the blood system to facilitate bidirectional communication between the lungs and the gut, exerting distinct effects on each organ. (4) Microbial components such as lipopolysaccharides are another soluble substance that facilitate communication between the lungs and the gut via the circulatory system. (5) Dysbiosis of gut microbiota is a visible manifestation of an imbalanced lung–gut axis, and changes in the composition and functions of the gut microbiota can influence the respiratory system via a shared mucosal immune system. LPS, lipopolysaccharide; SCFA, short‐chain fatty acid; TMAO, trimethylamine N‐oxide.

In brief, the homology between the lungs and intestines is the structural basis for the gut–lung axis. Immune cells and their interactions exert an essential role in the physiological process. Gut metabolites and microbial components, including LPS, serve as indispensable mediators in the gut–lung axis. In addition, changes in the composition and functions of the gut microbiota can influence the respiratory system via a shared mucosal immune system, while lung diseases also cause dysfunction in the intestinal tract. The knowledge of the gut–lung axis is briefly shown in Figure 2.

## GUT MICROBIOTA PROFILES OF PH

The gastrointestinal tract contains hundreds of microbial communities, which closely engage with their hosts and provide genetic, metabolic, and immunological benefits. Alterations and dysbiosis in the gut microbiota have been noted in diverse diseases, including CVDs, colon cancer, obesity, and rheumatoid arthritis. Based on the theoretical knowledge of the gut–lung axis, the association between gut microbiota and PH has been reasonably hypothesized, and extraordinary outcomes have been achieved in recent years. Alterations in gut microbiota have been observed in PH patients and animal models of PH induced by hypoxia or monocrotaline (MCT). Here, we summarize the knowledge of gut microbiota profiles in PH to lay the groundwork for a deeper comprehension of the “gut‐PH” axis (Table 1).

**Table 1:** Microbes detected in pulmonary hypertension.

References	Sample (sample size)	Differential taxa feature in PH
Kim et al. [28]	PAH patients (18) Healthy subjects (13)	**↑**: *Coprococcus*, *Butyrivibrio*, *Lachnospira*, *Eubacterium*, *Akkermansia*, *Bacteroides*, *Enterococcal*, *Bifidobacterium* ↓: *Lactococcal*
Jose et al. [110]	PAH patients (20) Healthy subjects (20)	**↑**: *Anaerostipes rhamnosivorans* **↓**: *Amedibacterium intestinale*, *Ruminococcus bicirculans*, *Ruminococcus albus*, *Lachnospiraceae bacterium* GAM79
Ikubo et al. [111]	CTEPH patients (11) Healthy subjects (22)	**↓**: *Faecalibacterium*, *Roseburia*, *Fusicatenibacter*, α‐diversity
Moutsoglou et al. [112]	PAH patients (72) Healthy subjects (39) Family subjects (15)	✓ **PAH patients vs. healthy subjects:** **↑**: *Bacteroides*, *Bacteroides thetaiotaomicron*, *Parabacteroides distasonis*, and *Bacteroides vulgatus* **↓**: Shannon diversity, Lachnospiraceae, *Faecalibacterium prausnitzii*, *Eubacterium rectale*, *R. bicirculans*, *Roseburia sp*., and *Bifidobacterium adolescentis* ✓ **PAH patients vs. family subjects:** ↓: Shannon diversity, *E. rectale*, *Butyrivibrio sp*., *B. angulatum*, *Lachnospira pectinoschiza*, and *R. torques*
Callejo et al. [113]	HySu rats (4) Control rats (4)	↑: Peptostreptococcaceae **↓**: Bacteroidetes (*Butyricimonas*, *Odoribacter*, *Porphyromonas*), *Cyanobacteria‐related bacteri*, *Acidobacteria*, *Butyricimonas*, *Odoribacter Porphyromonas*
Sanada et al. [114]	HySu rats (4) Control rats (6)	**↑**: *Rothia*, Prevotellaceae, *Parabacteroides*, *Parasutterella*, *Allobaculum*, *Parvibacter*, *Faecalibaculum*, Ruminococcaceae, *Bifidobacterium*, Lachnospiraceae, *Eubacterium coprostanoligenes* group, *Coprococcus 3*, *Acetitomaculum* **↓**: Firmicutes, Actinobacteria, Cyanobacteria, *Bacteroides*, *Akkermansia*, *Dehalobacterium*, *Marvinbryantia*, *Enterococcus*, Bacteroidetes S24‐7 group uncultured bacterium
Sharma et al. [115]	Hypoxia mice (8) Control mice (7)	**↑**: Chao 1 richness, Shannon diversity, evenness, *Prevotella*, *Oscillospira*, *Ruminococcus* **↓**: F/B ratio, *Lactobacillus*
Sharma et al. [116]	MCT rats (6) Control rats (5)	**↑**: F/B ratio, Corynebacteriaceae, Erysipelotrichaceae **↓**: Enterobacteriaceae
Hong et al. [117]	MCT rats (6) Control rats (6)	**↑**: Firmicutes, Proteobacteria, Actinobacteria **↓**: Bacteroidota, Spirochaetota
Luo et al. [118]	Hypoxia rats (5) MCT rats (5) HySu rats (5) Control rats (5)	✓ **Hypoxia group:** ↑: Richness, Firmicutes **↓**: α‐diversity, Actinobacteria, unidentified bacteria, Verrucomicrobia ✓ **MCT group:** ↑: Richness, Firmicutes, Actinobacteria **↓**: Bacteroidetes, unidentified bacteria, Verrucomicrobia ✓ **HySu group:** ↑: Richness, Firmicutes **↓**: Bacteroidetes, unidentified bacteria
Nijiati et al. [119]	High‐altitude PH rats (10)Control rats (10)	**↑**: F/B ratio, Prevotellaceae, Desulfovibrionaceae **↓**: Lactobacillaceae, Lachnospiraceae
Chen et al. [120]	Left pulmonary artery ligation‐induced rats (11) Sham operation rats (11)	**↑**: *Sporobacte* **↓**: *Desulfovibrio*, *Eubacterium*

Abbreviations: CTEPH, chronic thromboembolic pulmonary hypertension; F/B ratio, Firmicutes‐to‐Bacteroidetes ratio; HySu, Hypoxia/Sugen 5416; MCT, monocrotaline; PAH, pulmonary arterial hypertension; PH, pulmonary hypertension.

### PH patients

#### PAH patients

A recent study demonstrated adult PAH patients had distinct gut microbiota profiles [28]. Compared to controls, PAH patients exhibited significantly decreased α‐diversity, bacterial richness, and evenness. Actinobacteria phylum, specifically the *Bifidobacterium*, was enriched in these patients, while propionate‐producing bacteria, including *Akkermansia* and *Bacteroides*, and butyrate‐producing bacteria, including *Butyrivibrio*, *Lachnospiraceae*, *Coprococcus*, and *Eubacterium* were decreased. Furthermore, functional alterations in the gut microbiomes were observed, including increased production of arginine, proline, and ornithine and increased groups of bacterial communities associated with TMA/TMAO and purine metabolism in PAH. Individuals with PAH also showed a higher prevalence of various species known for their pro‐inflammatory properties, such as *Bacteroides thetaiotaomicron*, *Parabacteroides distasonis*, and *Bacteroides vulgatus*, compared to healthy controls. Conversely, certain species with anti‐inflammatory attributes, such as *Faecalibacterium prausnitzii*, *Eubacterium rectale*, *Ruminococcus bicirculans*, *Roseburia* sp., *and Bifidobacterium adolescentis*), were observed to be lower in patients than in controls [112]. In a study by Jose et al., [110] which enrolled a higher proportion of idiopathic PAH patients, a decrease of *Amedibacterium intestinale*, *R. bicirculans*, *Ruminococcus albus*, *and Lachnospiraceae bacterium* GAM79 was observed in patients compared with the healthy individuals.

#### Other subtype patients

A study [111] focused on the gut microbiota profiles in CTEPH, another subtype of PH. The feces from 11 CTEPH patients and 22 healthy controls were used to explore the differences in gut microbiota by metagenomic shotgun sequencing. α‐Diversity was significantly decreased in patients with CTEPH compared to controls. Notably, the relative abundance of bacteria, including *Faecalibacterium*, *Roseburia*, and *Fusicatenibacter*, which possess a range of biological functions such as anti‐inflammatory properties to maintain gut homeostasis, decreased and negatively correlated with endotoxin levels in patients. In addition, a recent study [121]. explored the gut microbiome with PH in lowlanders and highlanders. Compared to controls, TMA‐producing species were increased, and α‐diversity of gut microbiota showed the opposite trend among PH patients living in the lowland. However, no difference in these gut microorganisms was identified among highlanders with PH. This work provided gut microbial target distinctions among PH patients in lowland and highland, and further investigation is needed to explore the potential microbiota‐dependent pathogenesis.

### Animal models

Gut microbiota dysbiosis, characterized by an imbalanced ratio of Firmicutes to Bacteroidetes (F/B), has been demonstrated in various animal models of PH, including MCT‐induced, Sugen 5416‐hypoxia (SuHx)‐induced, hypoxia‐induced, high‐altitude‐induced, and left pulmonary artery ligation‐induced models.

MCT‐induced PAH rats are commonly used in translational studies, and the changes in gut microbiota in this model have also been elucidated. Compared to healthy controls, MCT‐induced rats exhibited an increased F/B ratio, and several pathogenic species, including *Clostridium*, *Turicibacter*, and *Mollicutes* genera, were predominant [116]. Actinobacteria, Firmicutes, Proteobacteria abundance increased, and Spirochaetota, Bacteroidetes, unidentified bacteria, and Verrucomicrobi decreased [117, 118].

A taxonomy‐based analysis of SuHx rats revealed a threefold increase in the F/B ratio compared to controls. However, the altered ratio was primarily driven by a decrease in less abundant Bacteroidetes families, with no statistically significant alterations in the presence of Firmicutes families [113, 122]. In the genus level, compared with the control rats, the abundances of *Dehalobacterium*, *Marvinbryantia*, *Enterococcus*, *Akkermansia*, *Bacteroides*, and Bacteroidetes S24‐7 group uncultured bacterium were lower in SuHx rats, while 14 bacteria were significantly more abundant including *Acetitomaculum*, *Bifidobacterium*, *Faecalibaculum*, *Parvibacter*, *Allobaculum*, *Parasutterella*, *Parabacteroides*, and *Rothia* [114].

Similarly, an increased F/B ratio disrupted gut microbiota homeostasis in hyp‐induced PH mice. The study observed an enhancement in the abundance of harmful microorganisms, including Marinifilaceae, Lactobacillaceae, and Helicobacteraceae, while the beneficial Bacteroidaceae, Tannerellaceae, Prevotellaceae, and Lachnospiraceae were significantly decreased [123].

High‐altitude pulmonary hypertension (HAPH) is a disease that specifically affects populations living in high‐altitude areas. The pathogenesis of this disease involves the vasoconstriction and remodeling of pulmonary blood vessels, ultimately leading to high‐altitude heart disease. A study showed that there were alterations in the gut microbiota in a rat model of HAPH. The F/B ratio and abundance of Lactobacillaceae and Planococcaceae increased, and the abundance of Prevotellaceae and Desulfovibrionaceae at the family level decreased [119]. Furthermore, in the left pulmonary artery ligation‐induced PH rat model, researchers found that the richness of Sporobacteria increased while the abundance of Eubacteriaceae, Deltaproteobacteria, and *Desulfovibrio* conversely decreased, indicating a novel perspective on stress‐associated experimental PH [120].

**Figure 3:**
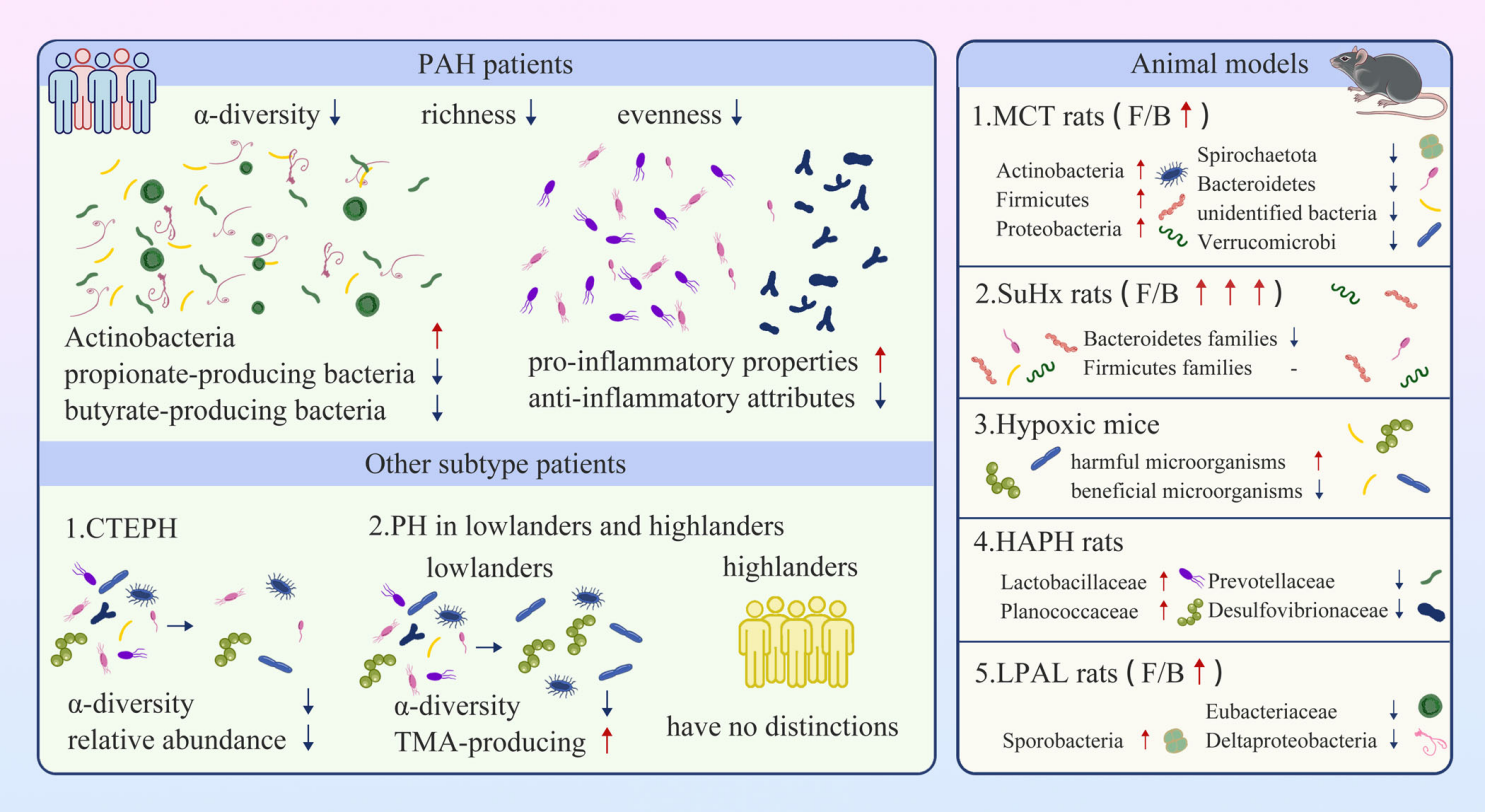
Distinct gut microbiota profiles of PH. PH is defined as an average pressure of greater than 20 mmHg in mean pulmonary artery pressure on supine right heart catheterization at rest. Compared to controls, PAH patients exhibited significantly decreased α‐diversity, bacterial richness, and evenness. Actinobacteria and microorganisms with pro‐inflammatory properties are increased while propionate‐ and butyrate‐producing bacteria are shown the opposite situation. α‐Diversity and the bacteria with anti‐inflammatory properties are significantly reduced in patients with CTEPH. Compared to controls, TMA‐producing species were increased, and α‐diversity of gut microbiota showed the opposite trend among PH patients living in the lowland, while no difference in these gut microorganisms was identified among highlanders with PH. Gut microbiota dysbiosis, characterized by an imbalanced ratio of Firmicutes to Bacteroidetes (F/B), has been demonstrated in various animal models of PH. The changes in gut microbiota may be involved in the pathogenesis of PH. CTEPH, chronic thromboembolic pulmonary hypertension; HAPH, high‐altitude pulmonary hypertension; LPAL, left pulmonary artery ligation; MCT, monocrotaline; PAH, pulmonary arterial hypertension; PH, pulmonary hypertension; SuHx, sugen 5416‐hypoxia; TMA, trimethylamine.

These pieces of evidence suggest the possible causative roles of microbiota changes in the early pathogenesis of PAH, while further investigations of their effects are warranted. The distinct gut microbiota profiles associated with PH are briefly demonstrated in Figure 3.

## GUT MICROBIOTA‐DEPENDENT METABOLITES IN PH

Regarding the alterations in the composition and function of the gut microbiota in PH, gut microbiota‐dependent metabolites have been a pivotal regulator ascribing to their local and systemic effects on host physiology or disease pathogenesis. Here, we summarized the knowledge of metabolites in PH based on the published studies, which provided an innovative paradigm for disease management and disease control.

### TMAO‐associated metabolites

To date, TMAO‐associated metabolites are the most explored gut microbiota‐associated molecules in the field of PH, mainly elucidated by our research group. TMAO has been fully investigated for its role in CVDs. TMAO has been proven to induce myocardial hypertrophy and fibrosis, promote endothelial cell and vascular inflammatory responses, induce cardiac mitochondrial dysfunction, and facilitate platelet reactivity and thrombus formation, directly impacting the blood vessels and heart and exacerbating the progression of cardiac disease [70, 124, 125, 126]. Our team has previously studied the association between TMAO with vascular damage and cardio‐cerebrovascular diseases, including vascular aging [36], chronic heart failure [127], cognitive impairment [35], stroke [37], amyotrophic lateral sclerosis [128], and type 2 diabetes [129].

Most importantly, for the first time, we have explored the effects of TMAO in PH patients, highlighting its potential biomarker role in disease management [40]. We selected the confirmed Group 1 PAH patients by right heart catheterization and comprehensive clinical examination. After reducing the bias through rigorous inclusion and exclusion criteria and statistical model construction, we analyzed the relation between circulating TMAO levels with PAH patients' disease conditions and prognosis. Our results showed that high TMAO levels were associated with severe disease conditions and poor prognosis manifested as an increased risk of heart failure and deterioration of PAH. In addition, our later study [130] further confirmed the potential biomarker role of plasma TMAO level in different PH subtypes, including idiopathic/heritable PAH and PAH associated with congenital heart disease, except for in CTEPH. It is speculated that TMAO may be related to the pathogenesis of PH, which has been verified in basic research. In an animal study, a TMAO inhibitor named 3,3‐dimethyl‐1‐butanol (DMB) enabled to ameliorate PH induced by MCT, accompanied by a decrease in abnormal apoptosis, excessive cell proliferation, and TGF‐β expression, and restoration of endothelial nitric oxide synthase [40]. Huang et al. [131] revealed that TMAO promoted the proliferation and migration of pulmonary artery smooth muscle cells by upregulating the production of inflammatory factors in macrophages such as Kng1, Cxcl1, Cxcl2, Cxcl6, and interleukin 6, ultimately leading to pulmonary vascular remodeling. DMB could suppress the chemokines and cytokines produced by macrophages to alleviate the progression of PH. However, a study by Videja et al. [132] drew an opposite conclusion, stating the protective effect of TMAO on mitochondrial energy metabolism in an MCT‐induced rat model. It reminds us that our knowledge of TMAO in the pathogenesis of PH is still insufficient, which underscores the need for enhanced collaborative research efforts to fully depict the whole molecular landscape to reconcile the conflicting evidence surrounding this issue. Our research group is devoted to providing more solid evidence to uncover the mysterious veil of TMAO in PH.

Moreover, the values of other TMAO‐associated metabolites, including L‐carnitine, choline, trimethyllysine, and betaine in PH management, are also worth exploring. Our research has revealed that high betaine levels are associated with poor prognosis of patients with PH. However, further investigation is required to uncover the specific mechanisms underlying this association [41]. Choline, which serves as a precursor of TMAO, is involved in various physiological processes. Previous studies have demonstrated its potential biomarker role in CVDs, including acute coronary syndrome [133, 134] and hypertension [135]. Our team is the first to investigate the association between circulating choline levels and PH patients' prognosis, and we found similar biomarker effects of the metabolite in PH field [136]. Additionally, our team has discovered and defined TMAVA as another TMAO‐associated metabolite [39]. We have found that elevated TMAVA is associated with an increased risk of cardiac mortality and transplantation in heart failure. It accelerated the progression of cardiac hypertrophy by inhibiting carnitine synthesis and subsequent fatty acid oxidation [38]. Currently, we are exploring the role and value of TMAVA in the field of PH, aiming to identify novel pathogenesis targets for disease cure.

### SCFAs

The gut microbiota can modulate the host immune system by releasing immunomodulatory bacterial metabolites, among which the most studied products are SCFAs, the predominant end‐products during the fermentation of nondigestible carbohydrates. The generation of SCFAs results from complicated interactions between diet and gut microbiota in the intestinal environment. SCFAs have been defined as natural ligands for free fatty acid receptors 2 and 3, expressed in various cell types, including enteroendocrine and immune cells, indicating the essential signaling roles between the host and gut microbiota [59, 137].

The associations between SCFAs and CVDs have been explored in the last decade. SCFAs, as key bacterial metabolites in host physiology, exert multiple effects on CVDs [59, 138]. Most SCFAs play positive roles in CVD pathogenesis, including atrial fibrillation [139], hypertension [23, 140], and atherosclerosis [141, 142] through mediating protection against inflammation, oxidative and mitochondrial stress, and over‐proliferation. SCFA‐producing bacteria are capable of maintaining gut microbial homeostasis. Butyrate activates peroxisome proliferator‐activated receptor‐γ, promoting the colonic epithelium toward β‐oxidation and the preservation of an anaerobic environment, fostering the prevalence of obligate anaerobes in the colon. Conversely, the lack of butyrate prompts increased expression of the inducible nitric oxide synthase and expansion of Proteobacteria, which are facultative anaerobes capable of nitrate respiration and are typically more abundant in disordered states [65].

Notably, the potential benefits of SCFAs for PH are highly encouraging, suggesting that SCFAs hold great promise for disease management and treatment. Most recently, Moutsoglou et al. have provided substantial evidence supporting the presence of gut dysbiosis in individuals with PAH and proposed a potential mechanism for the development of pulmonary vascular disease. Their study revealed a distinctive gut microbial signature in PAH patients, characterized by decreased levels of anti‐inflammatory SCFAs in the plasma [112]. In animal studies, intervention with butyrate attenuated hypoxia‐induced PH in rats, resulting in reduced right heart hypertrophy, decreased right ventricular systolic pressure, and improved pulmonary vascular remodeling. Moreover, the butyrate exerted anti‐inflammatory effects, leading to decreased accumulation of alveolar and interstitial lung macrophages [143]. Butyrate was found to induce differentiation of T‐regulatory cells through activating G protein‐coupled receptors and/or promoting epigenetic modifications and suppressing the NF‐κB pathway in macrophages, thereby mitigating inflammatory responses. Additionally, it promoted T‐cell differentiation to increase the production of anti‐inflammatory factors such as interleukin‐10 [144, 145]. Propionic acid enhances regulatory T‐cell populations [146], which offers protection against the development of PAH [147]. Valeric and butyric acids are potent inhibitors of HDAC, and patients with PAH exhibit reduced copies of the genes that encode the enzyme amidase for valerate and butyrate production [112]. Increased expression of HDAC6 is observed in pulmonary artery smooth muscle cells, which promotes their survival and proliferation. [112] HDAC3 can stimulate the expression of inflammatory genes by regulating NF‐kB activity and recruiting monocytes to inflammatory sites. Although the anti‐inflammatory SCFA acetate was not significantly decreased in patients with PAH [148], there was a significant reduction in the number of gene copies that encode the enzyme propionate coenzyme and transferase and acetate‐producing bacteria in patients with PAH [113, 149]. Moreover, SCFAs have been shown to play a critical role in regulating the expression of tight junction proteins, thereby improving intestinal barrier function in various disease models [150, 151, 152, 153]. Consequently, a decrease in SCFAs weakens the intestinal barrier function, promotes oxidative stress, and increases the likelihood of gut inflammation and leakage. This can lead to the release of inflammatory factors into the bloodstream, which can then circulate to the lungs and cause pulmonary vascular remodeling. It has been described that the reduction of SCFA leads to the development of PH, highlighting the potential of increasing SCFA levels as a novel treatment strategy for this disease. In PH mice, consuming a high‐soluble‐fiber diet can decrease the levels of disease‐related bacteria and methanol sulfate in the blood plasma. This is achieved by increasing the abundance of SCFA and propionic acid‐producing bacteria, resulting in lower hypoxia‐induced right ventricular systolic pressure and pulmonary vascular resistance. These changes are accompanied by a reduction in the proportion of mesenchymal macrophages, dendritic cells, and nonclassical monocytes [154]. To conclude, SCFAs are involved in the pathogenesis of PH, while elucidating specific mechanisms is warranted in future studies. Moreover, clinical cohort studies are necessary to identify the associations between circulating levels of SCFAs and patients' prognosis, as well as to explore their potential as biomarkers in PH management.

### Other metabolites

**Figure 4:**
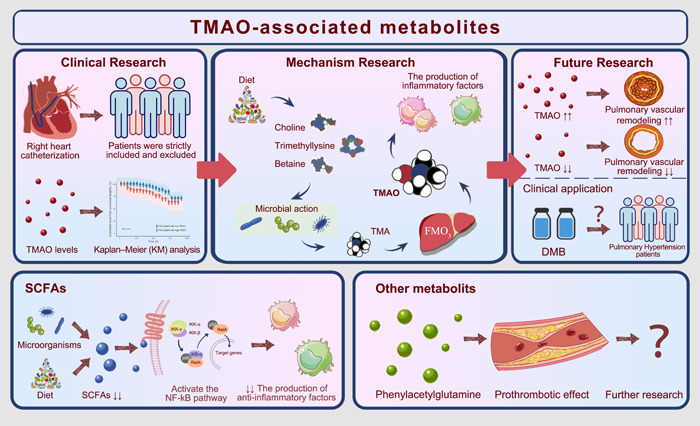
Gut‐microbiota associated metabolites in PH. TMAO is a potential biomarker in pulmonary hypertension. In our previous cohort study, patients were strictly included, and plasma TMAO levels were measured. High TMAO levels were associated with poor prognosis of patients with pulmonary hypertension. TMAO mainly stems from choline, abundant in red meat and fish. Intestinal flora choline‐TMA lyase can break it down to produce TMA, which enters the liver through the portal vein and is then oxidized by FMOs to generate TMAO ultimately. It has been elucidated that TMAO promoted pulmonary hypertension by upregulating the production of inflammatory factors in macrophages. In addition, a decrease in TMAO level by DMB indicates a preferable effect on pulmonary hypertension, and whether DMB can be used in clinics is worth further discussion. The generation of SCFAs results from complicated interactions between diet and gut microbiota in the intestinal environment. The SCFAs exert anti‐inflammatory effects, resulting in decreased accumulation of alveolar and interstitial lung macrophages. Decreased SCFAs activated the NF‐kB pathway and inhibited the production of anti‐inflammatory factors, which might promote the development of pulmonary hypertension. Phenylacetylglutamine is associated with atherosclerotic cardiovascular disease, the development of major adverse cardiovascular events, and to increase the risk of stroke through a potentially pro‐thrombotic effect. Whether phenylacetylglutamine is involved in the pathogenesis of PH, especially in subtypes associated with thrombosis, needs to be further investigated. DMB, 3,3‐dimethyl‐1‐butanol; FMO, flavin‐containing monooxygenases; NF‐kB, nuclear factor kappa‐B; PH, pulmonary hypertension; SCFA, hort‐chain fatty acid; TMA, trimethylamine; TMAO, trimethylamine N‐oxide.

Phenylacetylglutamine is a metabolite synthesized by both gut microbiota and host co‐involvement. It has been demonstrated in previous studies to be associated with atherosclerotic CVD, the development of major adverse cardiovascular events, and increasing the risk of stroke through a potentially pro‐thrombotic effect [32, 155, 156, 157]. It raises the question of whether phenylacetylglutamine is involved in the pathogenesis of PH, especially in subtypes associated with thrombosis. However, no study has clarified the role of phenylacetylglutamine in PH, which calls for further efforts in this field. In addition, the value of other metabolites in PH, including bile acids, branched‐chain fatty acids, and biogenic amine, is worth exploring to discover effective treatment modalities of PH. Figure 4 illustrates the knowledge of gut‐microbiota‐associated metabolites in PH.

## CONCLUSION

PH is a complex multifactorial disease characterized by increased pulmonary arterial pressure and vascular resistance leading to right heart failure and death. The intricate nature of the disease, which unfolds within the lung vasculature system, presents a challenging scenario for disease diagnosis and therapeutics. Consequently, the focus is shifting toward understanding the potential biomarkers and molecular targets for efficient prognosis and personalized therapy of PH.

Among these innovative approaches, the potential role and applicability of gut microbiome and its metabolites as novel biomarkers in the pathophysiology and treatment of PH cannot be undermined, as supported by our previous research findings. We have established a potential correlation between the gut microbiome and their metabolites in PH progression. The rapid advancements in large‐scale data technologies like artificial intelligence and machine learning hold enormous potential to revolutionize our understanding of PH. By harnessing these advanced technologies, we can effectively process and utilize complex big data derived from the gut microbiome to identify novel biomarkers, risk factors, and therapeutic targets. However, it is crucial to conduct long‐term longitudinal studies in the future to further validate the gut microbiome as a consistent and reliable biomarker of PH.

The gut microbiome plays a crucial role in modulating host immunity, inflammation, and metabolism, which can have significant implications for understanding PH. Through bacterial components and active metabolites, the gut microbiota interacts with the body's adaptive immune system, contributing to the establishment and maintenance of immune homeostasis [158]. Metabolites, in particular, have been identified as key mediators through which the gut microbiota can influence local and systemic immune responses, potentially contributing to the development of PH.

From this perspective, exploring the relationship between the gut microbiome, its metabolites, and PH might open up a new window for developing targeted and personalized solutions for PH. This shift from a “one‐size‐fits‐all” treatment paradigm toward a patient‐centered approach holds promise for more effective interventions. Various strategies, including fecal microbiota transplantation, probiotics, prebiotics, engineered microbiomes, and dietary interventions, can be explored as therapeutic targets to modify the pathogenesis of PH by modulating metabolites implicated in the disease.

**Figure 5:**
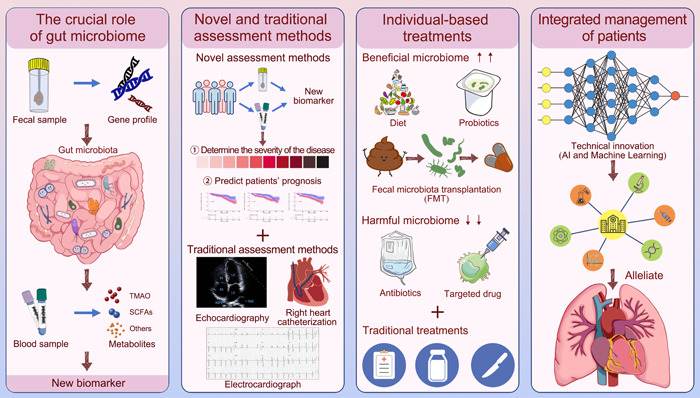
Future perspectives in pulmonary hypertension. Fecal and blood samples are utilized for metagenomic and metabolite exploration to discover new biomarkers in pulmonary hypertension. Effective biomarkers for assessing disease severity and prognosis will be developed and combined with traditional assessment methods, including echocardiography, right heart catheterization, and electrocardiogram, and they facilitate comprehensive assessment of disease condition. In addition to traditional treatments, increased beneficial microbiome through dietary intervention, probiotics, or fecal microbial transplantation and decreased harmful microbiome through antibiotics or targeted drugs are promising therapeutic strategies in pulmonary hypertension. Innovations powered by the rapid development of large‐scale data technologies like artificial intelligence and machine learning hold enormous potential to revolutionize our understanding of pulmonary hypertension. Leveraging these advanced technologies can facilitate the processing and application of complex big data from the gut microbiome, metabolites, clinical information, and therapeutic targets to achieve precise management of pulmonary hypertension.

Admittedly, translating these findings from research settings to clinical practice brings challenges. Ensuring appropriate microbiome alterations, monitoring treatment responses, and managing potential side effects are important considerations. Tailoring and personalizing these approaches based on an individual's unique microbiome composition and clinical needs might be a key to successful applications in the future. Figure 5 provides an overview of the potential applications of gut microbiota in PH patients.

In essence, exploring the gut–lung axis in PH opens up new horizons. It provides many opportunities for novel biomarker discovery, refined risk prediction, innovative therapeutic interventions, and more effective prevention initiatives. However, much remains to be learned about the complex interactions at the gut–lung interface. The future is undoubtedly ripe with opportunities for continued discovery and improved patient care.

## AUTHOR CONTRIBUTIONS

Yicheng Yang, Hanwen Zhang, and Jing Xu wrote the manuscript. Yaoyao Wang, Songren Shu, Peizhi Wang, and Shusi Ding revised the manuscript. Yaoyao Wang prepared the figures. Yuan Huang, Lemin Zheng, Changming Xiong, and Yuejin Yang supervised this project. All authors have read the final manuscript and approved it for publication.

## CONFLICT OF INTEREST STATEMENT

The authors declare no conflict of interest.

## Data Availability

This manuscript does not generate any code or data. Supplementary materials (graphical abstract, slides, videos, Chinese translated version, and update materials) may be found in the online DOI or iMeta Science http://www.imeta.science/.
